# Annotated compound data for modulators of detergent-solubilised or lipid-reconstituted respiratory type II NADH dehydrogenase activity obtained by compound library screening

**DOI:** 10.1016/j.dib.2015.12.019

**Published:** 2015-12-17

**Authors:** Elyse A. Dunn, Gregory M. Cook, Adam Heikal

**Affiliations:** a Department of Microbiology and Immunology, University of Otago, Dunedin 9054, New Zealand; b Maurice Wilkins Centre for Molecular Biodiscovery, The University of Auckland, Private Bag 92019, Auckland 1042, New Zealand

## Abstract

The energy-generating membrane protein NADH dehydrogenase (NDH-2), a proposed antibacterial drug target (see “Inhibitors of type II NADH:menaquinone oxidoreductase represent a class of antitubercular drugs” Weinstein et al. 2005 [1]), was screened for modulators of activity in either detergent-solublised or lipid reconstituted (proteolipsome) form. Here we present an annotated list of compounds identified in a small-scale screen against NDH-2. The dataset contains information regarding the libraries screened, the identities of hit compounds and the physicochemical properties governing solubility and permeability. The implications of these data for future antibiotic discovery are discussed in our associated report, “Comparison of lipid and detergent enzyme environments for identifying inhibitors of membrane-bound energy-transducing proteins” [2].

**Specifications table**

**Table:** 

Subject area	*Chemistry, Biology,*
More specific subject area	*Antibiotic discovery, drug discovery, microbiology*
Type of data	*Table*
How data was acquired	*Spectrophotometry using Varioskan*® *Flash (Thermo Scientific) 96-well plate reader.*
Data format	*Analysed and annotated with physicochemical properties*
Experimental factors	*Type II NADH dehydrogenase was purified and either remained in detergent solubilized form or reconstituted into proteoliposome form.*
Experimental features	*Spectrophotometric determination of NADH oxidation at 340 nm*
Data source location	*University of Otago, Dunedin, New Zealand*
Data accessibility	*Data in article*

**Value of the data**

• The respiratory Type II NADH dehydrogenase (NDH-2) is an antimicrobial drug target and these annotated drug screening data provide information that may be used for further antimicrobial drug development.
• These data provide the identity of compounds from three libraries that either inhibit or stimulate NDH-2 activity in both detergent-solubilsed and lipid-reconstituted forms.
• The list of compounds is further annotated with ‘Rule of Five’ properties that may be used to inform future antimicrobial drug discovery and development.

## 1 Data

These data are the annotated hits identified by screening both detergent-solubilised (DS) and lipid-reconstituted (LR) respiratory Type II NADH dehydrogenase. The table contains the library; compound name, number of H-bond donors/acceptors, molecular weight, lipophilicity (logP) and degree of inhibition/stimulation for DS and LR protein (Table 1).

## 2 Experimental design, materials and methods

Type II NADH dehydrogenase (NDH-2) was purified and reconstituted into proteoliposomes as described by Dunn et al. [2], [3]. Detergent-solubilised (DS) NDH-2 or lipid-reconstituted (LR) NDH-2 were screened by end point 96-well assay (340 nm, Varioskan® Flash Thermo Scientific, in the presence of 100 μM menadione and 20 μM test compound. The reaction was initiated using 200 μM NADH. Screening was carried out in technical triplicate against each compound library. NADH: menadione oxidoreductase inhibition/stimulation was calculated as a percentage of control activity for either DS or LR protein [2]. Compounds showing inhibition (≤70% control) or activation (≥130% control) in at least 2 of the 3 independent screening replicates were considered hits. Compound libraries screened were Library of Pharmacologically Active Compounds (LOPAC, SigmaAldrich), the Natural Products Set II (National Institutes of Health, USA) and the Quinolinequinone Library (Malaghan Institute of Medical Research, NZ) [4]. Hits were annotated with library of origin, chemical name and the drug-like properties governing *in vivo* absorption as described by the ‘Rule of Five’ namely, lipophilicity (logP), molecular weight, hydrogen bond donor and hydrogen bond acceptors [5]. Where necessary logP was predicted using ALOGPS software provided by the Virtual Computational Chemistry Laboratory [6].

## Figures and Tables

**Table 1:** Inhibitors/activators of detergent-solubilized (DS) or lipid-reconstituted (LR) NDH-2 activity, detected by screening compound libraries.

**Library**^a^	**Compound name**	**H-bond donors**^b^	**H-bond acceptors**^b^	**Molecular weight**	**LogP**^b^	**% control activity DS**^c^	**% control activity LR**^c^
LOPAC	I-OMe-Tyrphostin AG 538	3	5	437.19	2	40±6.7	41.9±27.0
LOPAC	Myricetin	6	8	318.24	0	42±14.2	29.9±22.4
LOPAC	Morin	5	7	302.24	2	32±31.2	18.7±12.1
LOPAC	Nordihydroguaiaretic acid (NDGA)	4	4	302.37	4	33±24.4	18.7±23.1
LOPAC	Reactive blue 2	5	11	840.11	1*	44±7.3	36.53±44.5
LOPAC	Tyrphostin AG 538	4	5	297.27	1	37±7.4	38.1±28.2
LOPAC	Tyrphostin AG 698	3	3	308.34	1	63±2.1	67.6±2.3
LOPAC	Tyrphostin AG 528	2	3	308.34	3	54±21.2	55.3±15.9
LOPAC	Indirubin-3′-oxime	3	2	277.28	2	50±12.1	47.6±25.6
LOPAC	Hispidin	3	5	246.22	0	48±3.2	53.7±19.4
LOPAC	Tyrphostin AG 537	6	6	448.44	1	22±18.4	57.0±16..9
LOPAC	2,6-Difluoro-4-[2-(phenylsulfonylamino) ethylthio]phenoxyacetamide (PEPA)	3	4	402.44	2	136±3.1	133.7±15.2
LOPAC	Caffeic acid phenethyl ester	2	4	284.31	4	43±9.0	N/A
LOPAC	Piceatannol	4	4	244.25	3	35±5.5	N/A
LOPAC	Tyrphostin AG 808	3	3	304.31	2	7±16.5	N/A
LOPAC	4-Chloromercuribenzoic acid	1	2	257.16	2*	48±0.3	N/A
LOPAC	WIN 62,577	1	1	438.58	5	142±1.8	N/A
LOPAC	MJ33	1	7	514.49	8*	149±22.7	N/A
LOPAC	Pentylenetetrazole	0	0	138.17	0	132±2.9	N/A
LOPAC	Kenpaullone	2	1	327.18	5	65±2.9	N/A
LOPAC	Pregnenolone sulphate sodium	0	5	418.53	1*	68±1.1	N/A
LOPAC	Phloretin	4	2	274.28	1	65±3.8	N/A
LOPAC	Farnesylthiosalicylic acid	1	2	358.55	7	61±1.4	N/A
LOPAC	Tyrphostin AG 555	3	3	322.37	3	53±1.2	N/A
LOPAC	Suramin hexasodium	6	23	1429.19	3*	61±10.0	N/A
LOPAC	Rottlerin	5	8	516.55	1*	57±6.1	N/A
NIH	6H-Benzofuro[3,2-c] [1] benzopyran-6-one, 3,9-dihydroxy-	ND	ND	268.23	ND	65±22.2	N/A
NIH	6H-Pyrido[4,3-b]carbazole, 5,11-dimethyl-	ND	ND	246	ND	65±28.1	N/A
NIH	Michellamine B diacetic acid salt	ND	ND	877	ND	54±31.2	N/A
NIH	[9,9′-bi-4H-Naphtho[2,3-b]pyran]-4,4′-dione, 2,2′,3,3′-tetrahydro-5,5′,6,6′,8,8′-hexahydroxy-2,2′,3,3′-tetramethyl-	ND	ND	547	ND	33±11.5	N/A
NIH	Mangostin	ND	ND	410	ND	19±15.3	N/A
Quinonolone Quinone	Compound 18b	ND	ND	375.46	ND	41±22.7	46±19.8
Quinonolone Quinone	Compound 19	ND	ND	377.48	ND	36±16.1	38±5.1

a Compound libraries were screened for inhibitors of NDH-2 NADH oxidation. Libraries screened included the Library of Pharmacologically Active Compounds (LOPAC), The NIH (National Institutes of Health) Natural Products Set II, and the Quinolinequinone Library.

b ND=value not determined.

c Data variation is expressed as standard deviation, N/A=not applicable.
