# Supplementary material for: Annotated compound data for modulators of detergent-solubilised or lipid-reconstituted respiratory type II NADH dehydrogenase activity obtained by compound library screening
Source: Data Brief. 2015 Dec 17;6:275–8. doi: 10.1016/j.dib.2015.12.019 (PMC4706612; doi:10.1016/j.dib.2015.12.019)
Supplement: Supplementary file 1 — Supplementary material [file mmc1.docx]

**Declaration of Conflicting Interests**

The authors declare no potential conflicts of interest with respect to the research, authorship and/or publication of this article.
